# What Should They Publish about Their Work and Needs?

**Published:** 1915-12-25

**Authors:** 


### What should they Publish about their Work and Needs?

*To the Editor of* The Hospital.

*To the Editor of* The Hospital.

Sir,—I am indebted to you for the able criticism which you publish upon my letter of December 18, and I hope you will allow me to say a few words thereon.

You accuse me of desiring to withhold from the public much information of an "interesting and reliable character." Nothing could be further from the truth. The point that I raised was the extent to which statistical information was of interest to the general public or a reliable guide to the distribution of charity; and I ventured to express a doubt as to whether any generous donors were influenced (for example) by statistical details of management expenditure, or had the expert knowledge to make fair deductions from them.

I fully agree that the success of the voluntary hospitals depends upon the extent to which we can interest the general public in their work, and inform them of its character, but I think we need not look further than the London Hospital to realise that simple facts are in this respect worth more than innumerable figures.

You claim that the growth of the King's Fund and the increase in the income of voluntary hospitals has been due to the provision of statistical information. No doubt it has been one of the causes in that it has enabled the King's Fund to exercise a wise control approved by the public, and to give what amounts to a guarantee that the hospitals are properly managed. But I fancy that Royal example, a wider publicity, and an awakening of the nation's conscience to the needs of the sick poor have been far more potent influences, and I doubt if the business man accustomed to the ingenuities of balance-sheets is prepared to place much reliance upon statistics.

The example of other charities which publish far less statistical information than hospitals, and yet receive an abundant harvest of legacies, subscriptions, and donations, seems to bear out this contention, but I notice that you receive this argument in silence.

With the large part of your criticism which refers to my personal position and the hospital which I serve I do not propose to deal. It does not appear to me to be a fair or proper method of dealing with a letter which was anonymous. The name and status of the author is a piece of confidential information in the hands of the Editor to which no reference should be made, and upon which no criticism should be founded.

The springs of action which move generous donors are obscure, little absolute proof can be adduced, but I am still of opinion that while the public desire and deserve the fullest and frankest information of the work of hospitals, it is not best presented in a statistical form. —I am, dear Sir, yours faithfully,

Assistant Secretary.

[Our correspondent is entitled to his opinion, but it is opposed to the evidence, which rests upon the facts as recorded in the progress made over a period of years. He seems to regard statistics as rubbish, and if he refers to the article in The Hospital (p. 227 of December 11) on which he based his first letter, he will see that it was fuller information, not merely statistics, which was advocated. The example of other charities does not help his argument. In 1894 the ordinary income received by missions exceeded the ordinary income of the hospitals. In the seventeen years—1897 to 1913—the income of the hospitals shows an increase during this period of 85 per cent., whereas the income of missionary societies only increased by 68 per cent., a remarkable fact. Further, in 1913 the hospitals' income showed a large increase compared with 1912, whereas that of orphanages, homes, and charities showed a material falling off, whilst the income of blind, and deaf and dumb institutions remained about stationary. We may mention further in this connection that some of the general charities have year by year improved their business methods, and have displayed readiness to render and supply fuller accounts. If "Assistant Secretary" will read our comments on his first letter again their careful re-perusal must reveal to him that we offered no criticism upon his personal position, which we do not even refer to, nor upon the hospital which he serves. Further, his anonymity was and is complete. Finally, the statistical form is not suitable for appeal literature, though it is the tersest and best way of giving in the smallest space the greatest number of useful facts, which makes the larger donors and the keenest experts regard it as having a distinct value in certain circumstances. We gave an instance on page 254 of The Hospital last week, of the value attached by large donors to accurate information and guidance. This week we have the pleasure to record another striking example of this in the case of Sir Ernest Cassel and his distribution of £50,000 amongst the hospitals. Large donors, when they want further information, naturally consult those who are able to give it independently, in the completest and most accurate form. —Ed. The Hospital.]

